# In search of biomarkers for low back pain: can traction therapy effectiveness be prognosed by surface electromyography or blood parameters?

**DOI:** 10.3389/fphys.2023.1290409

**Published:** 2023-12-08

**Authors:** Marzena Ratajczak, Małgorzata Waszak, Ewa Śliwicka, Michał Wendt, Damian Skrypnik, Jacek Zieliński, Piotr Krutki

**Affiliations:** ^1^ Department of Medical Biology, Poznan University of Physical Education, Poznan, Poland; ^2^ Department of Physiology and Biochemistry, Poznan University of Physical Education, Poznan, Poland; ^3^ Department of Treatment of Obesity, Metabolic Disorders and Clinical Dietetics, Poznan University of Medical Sciences, Poznan, Poland; ^4^ Department of Athletics, Strength and Conditioning, Poznan University of Physical Education, Poznan, Poland

**Keywords:** lumbar traction, interleukin-2, interleukin-4, interleukin-10, interleukin-17A, stem cell growth factor

## Abstract

**Background:** Lumbar traction therapy is a common method to reduce low back pain (LBP) but is not always effective. The search for biomarkers that would prognose the effectiveness of LBP management is one priority for improving patients’ quality of life.

**Objectives:** 1) To determine the phenotype of patients benefiting most from lumbar traction therapy. 2) To correlate systemic and electromyographic biomarkers with pain and pain-related disability.

**Methods:** Data on muscle bioelectrical activity (surface electromyography [SEMG]) in the flexion-extension task, the concentrations of twelve systemic biochemical factors, LBP intensity (Visual Analog Scale), the Oswestry Disability Index, and the Roland–Morris Disability Questionnaire (RMDQ) were collected before and 72 h after 20 sessions of lumbar traction therapy. Patients were divided into responders and nonresponders based on the criterion of a 50% reduction in maximal pain.

**Results:** The responders had lower maximal muscle bioactivity in the extension phase on the left side (*p* < 0.01) and higher flexion-extension ratios on both sides of the body in the SEMG (left: *p* < 0.05; right: *p* < 0.01), and higher adipsin, interleukin-2, interleukin-4, and interleukin-10 concentrations (*p* < 0.05) than nonresponders. Patients with higher interleukin-4 concentrations before therapy achieved greater reductions in maximal pain in the sitting position, bioelectrical muscle activity in flexion, and flexion-relaxation ratio on the left side of the body. Changes in adipsin and interleukin-4 concentrations correlated with changes in LBP intensity (r = 0.68; r = −0.77). Changes in stem cell growth factor and interleukin-17A correlated with changes in RMDQ (R = 0.53) and bioelectrical muscle activity in extension (left: R = −0.67; right: R = −0.76), respectively.

**Conclusion:** Responders to traction therapy had SEMG indices of less favorable muscle activity in the flexion-extension task and elevated indices of inflammation before the study. For the first time, interleukin-4 was indicated as a potential biomarker for prognosing post-therapy changes in pain intensity and muscle activity.

## Introduction

The complex anatomy of the lumbar spine, where each structure can be affected by a variety of stressors, reflects difficulties in low back pain (LBP) diagnosis and treatment. The multitude of factors as well as the fact that they can overlap, combined with the low specificity of the diagnostic methods, cause that in many cases LBP management is mainly focused on reducing pain. Furthermore, poor diagnosis might be burdened with the risk of chronicization, overuse of imaging, opioids, and surgery. The search for new accurate and objective methods to examine the etiology of back pain is currently one priority for reducing the cost of treating LBP and improving patients’ quality of life (Knezevic et al., 2021).

One common method to reduce LBP is lumbar traction therapy, which decompresses the intervertebral discs and eliminates tissue pressure on the nerve roots. Studies on the method’s effectiveness have reported divergent results, mainly due to the heterogeneous samples of volunteers (Alrwaily et al., 2018). While patients share LBP, their symptoms may result from various structural or functional changes, e.g., discopathies, spinal stenoses, dysfunction of thoracolumbar fascia, or diseases, e.g., ostheorthritis, or metabolic disorders, e.g., hyperlipidemia, diabetes (Tarabeih et al., 2022). Inconsistent traction therapy results are also due to different traction modalities (intermittent vs. continuous) and loads (usually between 10% and 50% of body weight) (Alrwaily et al., 2018).

The greatest benefits of traction therapy are achieved by patients with disc herniation, with or without sciatica (Isner-Horobeti et al., 2016; Karimi et al., 2017; Masood et al., 2022). This outcome is associated with the discopathy formation process in which cells synthesize and release neurogenic factors initiating the growth of vessels and nerve fibers into the disc tissue (García-Cosamalón et al., 2010). Increased vascularity in a deteriorating disc can lead to changes in isolated degenerated intervertebral discs, coinciding with high cytokine concentrations (Kang et al., 1997). Moreover, structural remodeling and undesirable hyperstimulation of nociceptors were observed due to the infiltration of granulation tissue from the nucleus pulposus to the annulus fibrosus (Peng et al., 2005). All these factors contribute to pain. It is assumed that decompressing the discs and releasing the spinal nerve roots from compression following traction therapy allows the tissues to return to homeostasis, including the healing of inflammation in the degenerated or injured spinal or paraspinal tissues.

This observational study on women with chronic LBP undergoing lumbar traction therapy had two aims. The first was to determine the phenotype of patients benefiting most from lumbar traction therapy, which would allow only those patients who respond to traction therapy to be referred for it. The second was to examine the associations of systemic and electromyographic biomarkers with pain and pain-related disability. Finding relationships between blood concentrations of biochemical markers, pathoanatomical factors, and subjective and objective functional indicators would contribute to a better understanding of pain mechanisms in the spine. Because variables related to inflammation are affected by many factors related to age and sex, this study was intentionally conducted in a homogeneous group of premenopausal women of similar age, distinguishing it from previous studies of this type that were performed in the general population (Weber et al., 2015; Brady et al., 2018; Teodorczyk-Injeyan et al., 2019; Schaaf et al., 2021).

## Materials and methods

### Study design

**FIGURE 1:**
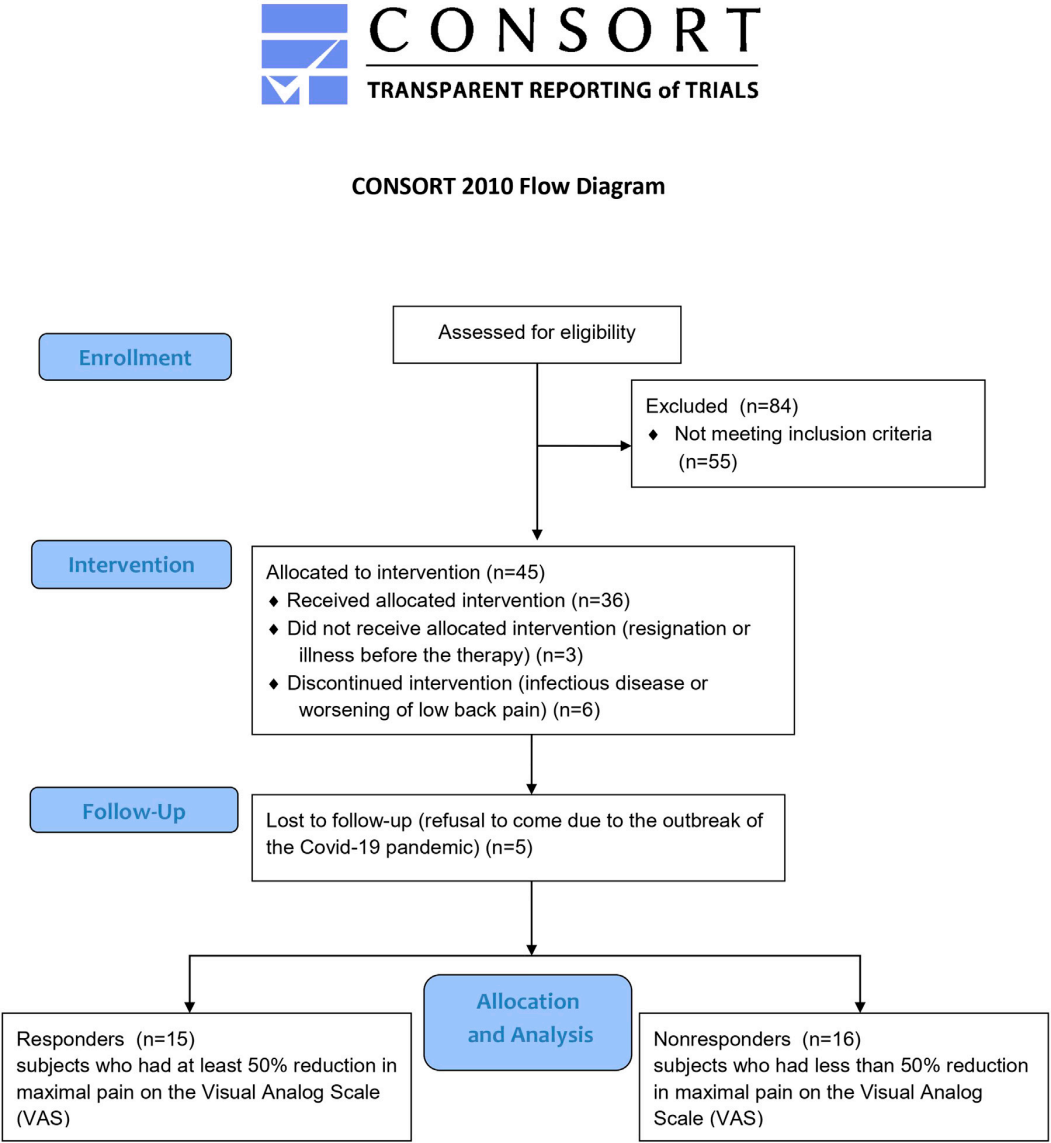
CONSORT flow diagram.

This study used data from a clinical trial conducted between August 2020 and May 2022 and registered in the ClinicalTrials.gov database (NCT04507074). This study adhered to the standards laid down in the Declaration of Helsinki. The study research protocol was approved by the Ethics Committee at the Poznan University of Medical Sciences in Poland (ref. 958/19), and all subjects provided informed consent before study participation. Women who received therapy and were not lost to follow-up in the clinical trial were merged into one study group, then divided into two groups (responders and nonresponders) based on their response to traction therapy. Participants underwent twenty systematically applied sessions of lumbar traction on a special split table (Therapy Traction Couches and Packages, ST6567P; SEERS Medical, UK), stretching the treated section of the trunk. The application of traction forces lasted 30 min daily, 5 days a week, for 4 weeks (20 therapeutic sessions). A constant (continuous) traction mode was used at the force level of 25% of the patient’s body weight during the first five treatment sessions and gradually increased to 30% of the body weight. A certified physiotherapist administered the therapy in the treatment room at Poznan University of Physical Education. All patients were instructed to maintain their normal physical activity and diet. Therapy responders were defined as those subjects who had a 50% reduction in maximal pain on the Visual Analog Scale (VAS) and whose degree of disability on the Oswestry Disability Index (ODI) or Roland–Morris Disability Questionnaire (RMDQ) had not worsened at clinical follow-up; otherwise, the subjects were categorized as nonresponders. The 50% reduction was chosen to be much higher than the 30% threshold that was considered a clinically meaningful improvement in subjective pain perception in a literature review (Ostelo et al., 2008), and it let us divide participants into similarly sized groups (Figure 1). At baseline, anthropometric and subjective pain-related measurements were performed, and blood samples were taken for laboratory analysis.

## Patients

Forty-five patients with chronic LBP and various degrees of intervertebral disc degeneration, aged 33–51 years met the inclusion criteria and thirty-six completed a cycle of 20 units of traction therapy. Women were recruited through social media. The inclusion criteria for the study were: women aged 30–52  years with chronic LBP lasting at least 6 months that was more severe than pain in any other part of the body; and stable body weight for the last month (±2 kg).

However, patients were excluded if they were pregnant or had: menopause, pain located elsewhere if stronger than the LBP, pathologies and/or medications that might affect the balance between pro- and anti-inflammatory factors (e.g., inflammatory disease, rheumatoid arthritis, ankylosing spondylitis, systemic lupus, acute infection, cancer, overt inflammatory process of the respiratory tract, genitourinary system or within head and neck, and alcohol abuse), type II diabetes, poorly controlled arterial hypertension, lipid disorders requiring pharmacological treatment in the last 3 months, chronic kidney disease, clinically significant impairment of liver function, acute coronary event, unstable angina, stroke or transient ischemia in the last 6 months, signs of heart failure, clinically significant arrhythmias or conduction disturbances, pacemaker implantation, serious neurological disorders, previous surgery, post-accident mechanical injuries in the area of the spine, medical diagnosis of spondylolisthesis, osteoporosis, history of syncope, uncontrolled mental illness, and other conditions that might pose any risk to the patient during the lumbar traction therapy.

## Measurements

### Discs degeneration

Before the intervention, the patients underwent magnetic resonance imaging (1.5T) to assess the degree of structural damage within intervertebral discs. Disc degeneration was assessed with the Modified Pfirrmann Grading System for Lumbar Intervertebral Disc Degeneration (Griffith et al., 2007). Intervertebral discs from Th12/L1 to L5/S1 were assessed in all patients by the same experienced radiologist. The system comprises eight grades, where one represents no disc degeneration (uniformly hyperintense signal from the nucleus and inner fibers of the annulus, equal to cerebrospinal fluid and normal disc height), and eight represents end-stage degeneration (hypointense signal and >60% reduction in disc height). The results represent the sum of scores for the six discs between Th12-S1.

All examinations listed below were performed at the Poznan University of Physical Education before and on the third day after the intervention.

### Body mass index and total body fat content

Anthropometric measurements were performed in the morning, with light clothing and without shoes. Body weight and height were measured using a medical scale with a stadiometer (Seca 285, Germany) to the nearest 0.1 kg and 0.5 cm, respectively. The BMI was calculated using the standard formula based on weight and height. Body composition was assessed using dual-energy X-ray absorptiometry (Lunar Prodigy device; GE Healthcare, IL, United States). Total body fat content was determined using the standard scan mode (for normal-weight and moderately obese subjects) or the thick scan mode (for extremely obese subjects); the absorbed radiation doses were 0.4 and 0.8 µGy, respectively.

### Low back pain severity

A 10 cm VAS was used to evaluate the pain severity 2 days before and 3 days after 20 sessions of the therapy. The patients were asked to mark the minimum or maximum score corresponding to their pain level on the pain scale, in the previous week which was between 0 (no pain) and 10 (the worst pain imaginable). The scale was used in four categories: the maximum morning pain, the maximum night pain, the maximum pain while sitting, and the maximum pain while standing. The VAS score has already been used in patients with LBP undergoing traction therapy (Liu et al., 2021) and appears reliable in assessing pain severity (Shafshak and Elnemr, 2021).

### Low back pain-related functionality

Patient disability and functionality were assessed using the ODI and RMDQ. Both questionnaires are reliable and valid for measuring disability or functional level in Polish-speaking patients with LBP (Opara et al., 2006; Miekisiak et al., 2013).

### Biochemical analysis

Blood samples for biochemical analyzes were taken twice: before the intervention, and 72 h after the last session of the therapy. Serum was taken from a basilic vein after overnight 12-h fasting. The patients were asked not to take anti-inflammatory drugs for at least 48 h before the blood sampling. We focused on the following biomarkers which role is discussed in the literature in the context of LBP: the aggrecan chondroitin sulfate 846 epitope (CS846), which is present in the plasma of people with osteoarthritis (Kong et al., 2006), adipsin and leptin which are found in higher concentrations in people with back pain (Brady et al., 2018), neuropeptide Y which is a pain protective factor secreted by cells subjected to inflammatory stress (Dombrowski et al., 2020), vascular endothelial growth factor A (VEGF-A), stem cell growth factor (SCGF) and interleukins 2 (IL-2) and 17A (IL-17A), the concentrations of which were reduced as a result of therapy in patients with intervertebral disc disorders (Weber et al., 2015), regulated on activation in normal T-cell expressed and secreted (RANTES) concentration, which correlates with pain perception in patients with LBP (Sowa et al., 2014), growth/differentiation factor 15 (GDF-15), which has a strong correlation with LBP associated disability (Tarabeih et al., 2019), and interleukins 4 (IL-4) and 10 (IL-10), concentrations of which may have an analgesic effect, what was hypothesized in research on sciatica (Wang et al., 2016). Parameters were measured in the serum samples using commercially available enzyme-linked immunosorbent assays (ELISAs). CS846 was assessed using the test made by IBEX Pharmaceuticals Inc. (Canada). Adipsin and neuropeptide Y, VEGF-A, RANTES, and SCGF were analyzed using an ELISA kit from Cloud-Clone Corp. (TX, United States). Leptin and GDF-15 were measured using tests made by BioVendor Research and Diagnostic Products (The Czech Republic). IL-2, IL-17A, IL-4, and IL-10 were measured using tests made by SunRed Biotechnology Company (China). Additionally a ratio between proinflammatory IL-2 and anti-inflammatory IL-10 was calculated as it was described by Teodorczyk-Injeyan et al., who observed differences in this ratio between LBP patients and asymptomatic individuals (Teodorczyk-Injeyan et al., 2019).

### Surface electromyography in the flexion-extension task

**FIGURE 2:**
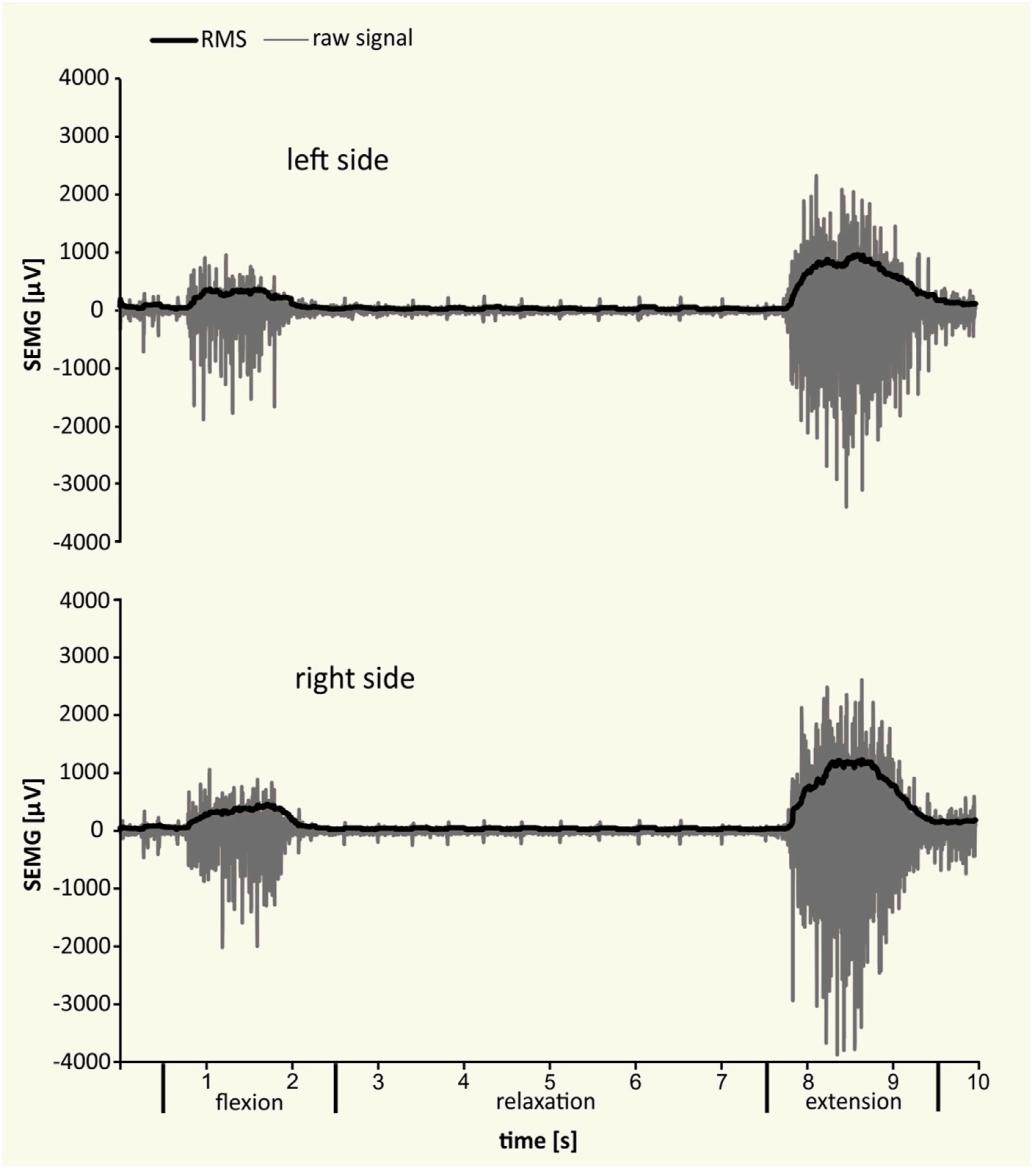
Examples of SEMG recordings from left and right longissimus muscles during standing, trunk flexion, relaxation, and extension. The raw signal and the RMS of bioelectrical activity are presented.

SEMG was carried out in the morning, in a separate room, with an electromyographic system with plate electrodes (model W4X8; Biometrics Ltd., United Kingdom). The results were recorded using DataLog Bluetooth V7.5 software (Biometrics Ltd., United Kingdom). During the examination, two surface electrodes (type SX230 1000) were attached with adhesive tape after disinfection and wiping the skin a few times with salicylic alcohol to reduce its resistance. The reference electrode (type R230; Biometrics Ltd., United Kingdom) was fixed at the distal end of the radius (Lister’s tubercle region) with an elastic band. The examination involved the lumbar segment of the longissimus muscle, both right- and left-sided bundles. The electrodes were placed according to the international guidelines published by the Surface ElectroMyoGraphy for the Non-Invasive Assessment of Muscles project (The SENIAM project et al., 2018) on the erector spinae muscle at L2 level. The two recording electrodes were placed about 3 cm horizontally from the midline. A 10-min warm-up of the key muscle groups preceded the measurement. Subjects were asked to perform a standing trunk flexion and extension task while the SEMG was recorded (Figure 2). This task lasted about 10 s and was divided into four phases: standing (the patient was standing without movement with their feet at hip width), flexion (the patient bent forward with a slow and controlled movement to reach maximal trunk flexion), relaxation in full flexion (the patient relaxed the muscles during the full flexion position), and extension (the patient returned to the upright posture). Each patient repeated the task after completing a trial at least once. The list of determined electromyographic parameters included the maximal and mean values of the root mean square (RMS) of a bioelectrical signal (maximal bioelectrical activity [MBA]) from the longissimus muscle in the flexion, relaxation, and extension phase.

Flexion-relaxation ratio (FRR) (Neblett et al., 2013) and flexion-extension ratio (FER) (Wei et al., 2019) were calculated from the above data according to the following formulas:

$$FRR=\frac{\text{maximum}\;\text{SEMG}\;\text{values}\;\text{during}\;\text{flexion}}{\text{mean}\;\text{SEMG}\;\text{values}\;\text{during}\;\text{maximum}\;\text{voluntary}\;\text{flexion}\;\left(\text{relaxation}\;\text{phase}\right)}$$

$$FER=\frac{\text{maximum}\;\text{SEMG}\;\text{in}\;\text{flexion}\;\left(\text{relaxation}\;\text{phase}\right)}{\text{maximum}\;\text{SEMG}\;\text{during}\;\text{extension}}$$

### Statistical Analyses

In order to confirm that the number of patients divided into responders and nonresponders is sufficient to show differences in the concentration of the most expected changes in the ANOVA test, the sample size was calculated based on our previous study with two groups of chronic LBP patients (*n* = 13 and *n* = 15) who received traction therapy with the same settings and modality (Ratajczak et al., 2023). GDF-15 was the variable in which we expected differences, therefore we took data on means, standard deviations and correlation coefficients among repeated measures from the previous study before and after the therapy and from both studied groups. Then, the power analysis was performed using repeated measures analysis of variance (ANOVA) between factors in G*Power 3.1.9.4. The effect size was determined from the partial ƞ^2^ (0.405). Analysis indicated that a minimum of eight subjects per group was required to provide at least 80% power of detecting an intervention effect as statistically significant at α = 0.05.

Data analyses were performed using the Statistica 13.3 software package (TIBCO Software Inc.; Palo Alto, CA, United States). Data are presented as mean (standard deviation [SD]). The normal distribution of variables was confirmed with the Shapiro–Wilk test. The differences in means between the responder and nonresponder groups were assessed using the non-parametric Mann–Whitney U test for variables with a non-normal distribution and heterogeneous variances and Student’s t-test for variables with a normal distribution and homogeneous variances. A two-way repeated measures ANOVA was used to assess the effects of time, group, and the time × group interaction. Tukey’s honestly significant difference (HSD) with unequal N *post hoc* test was used to assess the significance of differences between pairs of measurements. The eta squared coefficient (η^2^) is presented as an effect size indicator. Correlation analyses used *Pearson’s correlation* coefficient and Spearman’s rank correlation coefficient.

## Results

Before traction therapy, the concentrations of adipsin, IL-2, Il-4, and Il-10 were significantly higher in responders than in nonresponders. However, the ratio of proinflammatory interleukin-2 to anti-inflammatory interleukin-10 was similar in responders and nonresponders (*p* = 0.44186). The responders also had lower maximal muscle bioelectrical activity in the extension phase on the left side and higher FER values on both sides of the body in the SEMG test (Table 1).

**TABLE 1:** Baseline clinical measurements for responders (>50% pain relief) and nonresponders.

Measure	Responders (n = 15) mean (SD)	Nonresponders (n = 16) mean (SD)	*p*-value
**Age [years]**	42.4 (5.29)	41.81 (4.32)	0.73662^a^
**Body mass index [kg/m** ^**2**^ **]**	31.45 (6.19)	27.27 (5.69)	0.08559^b^
**Total fat content [%]**	43.19 (7.16)	39.51 (6.70)	0.14932^a^
**Maximal pain [VAS 0–10]**	5.08 (2.44)	5.96 (2.66)	0.34443^a^
**Chronic LBP duration [years]**	9.75 (7.94)	11.14 (7.89)	0.64551^a^
Lumbar discs degeneration [8–48]*	16.66 (5.38)	15.38 (4.63)	0.47865^a^
**RMDQ [0–24]**	4.66 (3.62)	4.31 (2.89)	0.76480^a^
**Oswestry Disability Index [0–50]**	11.93 (5.99)	12.06 (5.59)	0.95092^a^
**Beck Depression Inventory (0–63)**	6.53 (5.22)	8.00 (6.95)	0.51390^a^
**CS846 [ng/mL]**	21.54 (3.56)	20.81 (2.85	0.48910^b^
**Neuropeptide Y [pg/mL]**	738.14 (253.41)	680.28 (204.84)	0.48873^a^
**Leptin [ng/mL]**	30.89 (16.44)	23.08 (13.28)	0.15510^a^
**Adipsin [ng/mL]**	8.26 (1.23)	7.07 (1.65)	0.03038^a^
**GDF-15 [pg/mL]**	483.48 (222.58)	490.93 (240.61)	0.95272^b^
**VEGF A [pg/mL]**	21.54 (5.32)	29.03 (24.87)	0.44082^b^
**SCGF [ng/mL]**	33.23 (32.76)	28.76 (6.95)	0.35293^b^
**RANTES [ng/mL]**	6.32 (1.26)	6.12 (0.53)	0.98423^b^
**Interleukin-2 [pg/mL]**	16.96 (18.72)	8.15 (4.14)	0.02615^b^
**Interleukin-4 [pg/mL]**	53.89 (29.73)	30.21 (11.41)	0.01582^b^
**Interleukin-10 [pg/ml]**	7.72 (2.25)	5.66 (2.10)	0.01331^a^
**Interleukin-17A [pg/mL]**	52.48 (55.97)	26.69 (11.78)	0.10176^b^
**MBA flexion—L [μV]**	1195 (818)	1235 (777)	0.89049^a^
**MBA flexion—R [μV]**	1427 (811)	1274 (683)	0.57248^a^
**MBA relaxation—L [μV]**	241 (173)	184 (157)	0.27702^b^
**MBA relaxation—R [μV]**	236 (151)	171 (148)	0.14359^b^
**MBA extension—L [μV]**	997 (829)	1262 (338)	0.00962^b^
**MBA extension—R [μV]**	981 (480)	1252 (627)	0.19891^b^
**FRR—L**	17.53 (25.63)	15.01 (10.85)	0.46461^b^
**FRR—R**	17.36 (14.77)	31.52 (49.11)	0.54008^b^
**FER—L**	0.31 (0.25)	0.17 (0.17)	0.03122^b^
**FER—R**	0.27 (0.18)	0.17 (0.20)	0.00962^b^

* The subjects had various degrees of intervertebral disc degeneration.

^a^ Unpaired *t*-test.

^b^ Mann-Whitney U test; LBP, low back pain; RMDQ, Roland-Morris Disability.

Questionnaire; CS846, aggrecan chondroitin sulfate 846 epitope; GDF-15, growth and differentiation factor 15; VEGF, vascular endothelial growth factor; SCGF, stem cell growth factor; RANTES, regulated on activation, normal T-cell expressed and secreted; MBA, maximal bioelectrical activity of the longissimus muscle; FRR, flexion-relaxation ratio; FER, flexion-extension ratio; L, left side; R, right side.

**FIGURE 3:**
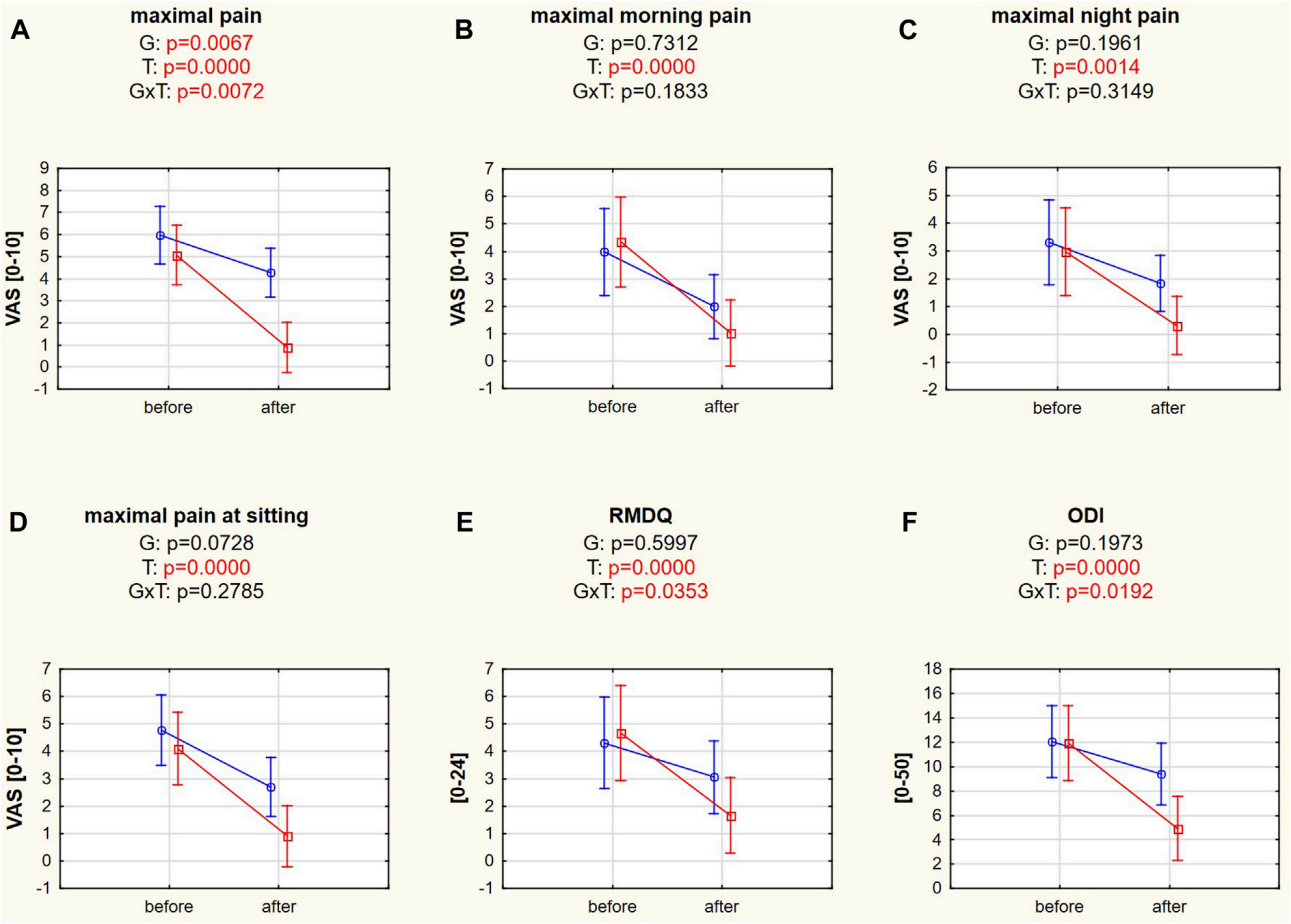
**(A–F)** Pain intensity and disability variables in responders (red square-ended line; >50% pain relief) and nonresponders (blue circle-ended line; <50% pain relief) before and after therapy. G refers to the group effect, T refers to the time effect, and GxT refers to the interaction in a two-way ANOVA.

Maximal LBP differed significantly between groups and examination dates, confirmed by Tukey’s *post hoc* HSD tests (*p* = 0.0068 and *p* = 0.0001), and proved to be a reliable variable for dividing patients into responders and nonresponders. The *post hoc* test for interaction also confirmed the different responses to treatment in the responder (*p* = 0.0002) and nonresponder (*p* = 0.0433) groups, which did not differ significantly at baseline (*p* = 0.7274; Figure 3A). Maximal morning pain, night pain, and maximal pain while sitting were significantly reduced after treatment (*post hoc* tests: *p* = 0.0002, *p* = 0.0016, *p* = 0.0002, respectively; Figures 3B–D). Similarly, disability levels decreased in the RMDQ (*post hoc* test: *p* = 0.0002) and ODI (*post hoc* test: *p* = 0.0001). However, significant differences in RMDQ and ODI over time were found only in the responder group (*post hoc* tests: *p* = 0.0002, *p* = 0.0002, respectively; Figures 3E,F, Supplementary Table S1).

**FIGURE 4:**
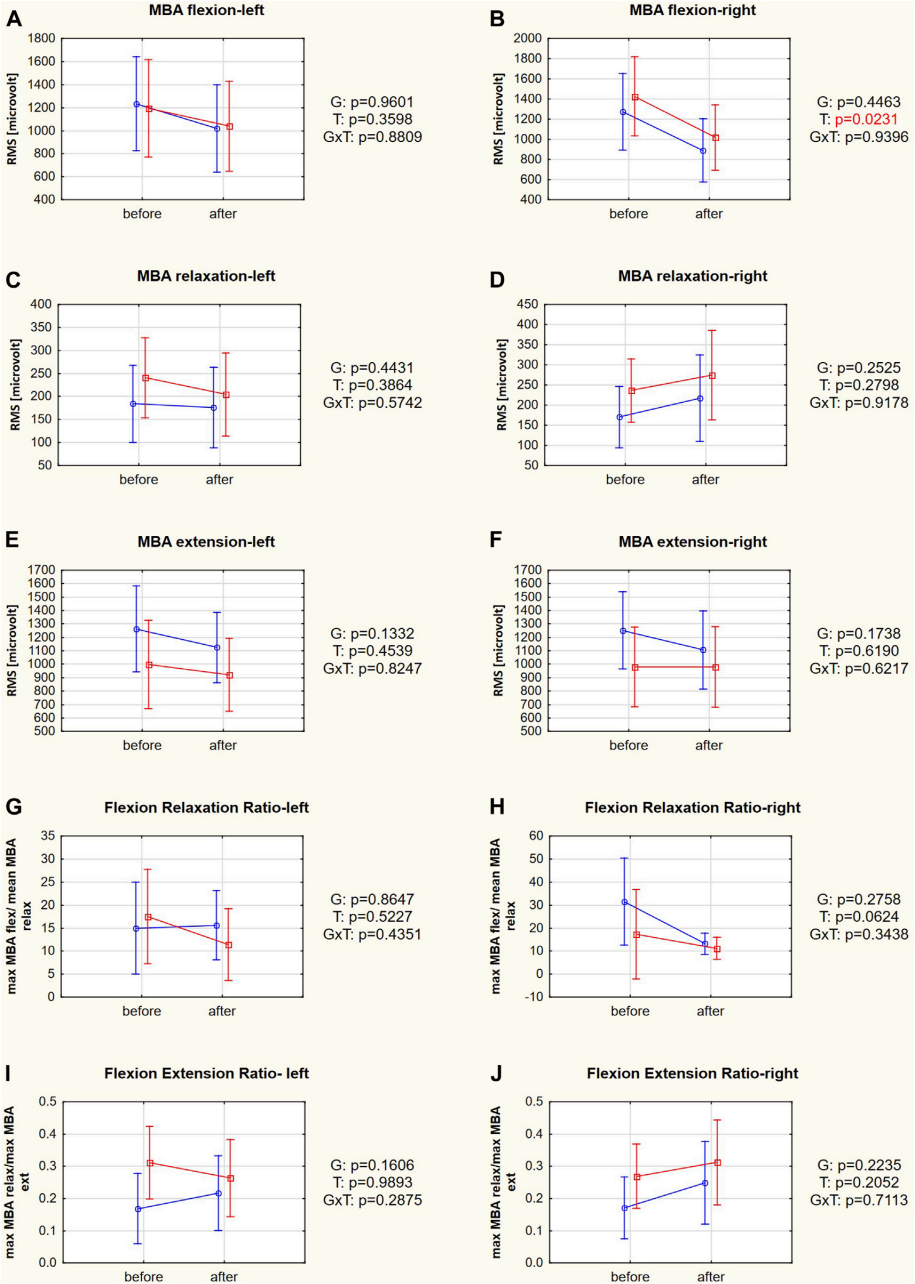
**(A–J)** SEMG variables in responders (red square-ended line; >50% pain relief) and nonresponders (blue circle-ended line; <50% pain relief) before and after therapy. G refers to the group effect, T refers to the time effect, and GxT refers to the interaction in a two-way ANOVA.

The only SEMG variable that changed significantly after the therapy was MBA flexion on the right side (*post hoc* test: *p* = 0.0233; Figure 4B). Other SEMG variables did not improve due to the therapeutic intervention (Supplementary Table S2).

**FIGURE 5:**
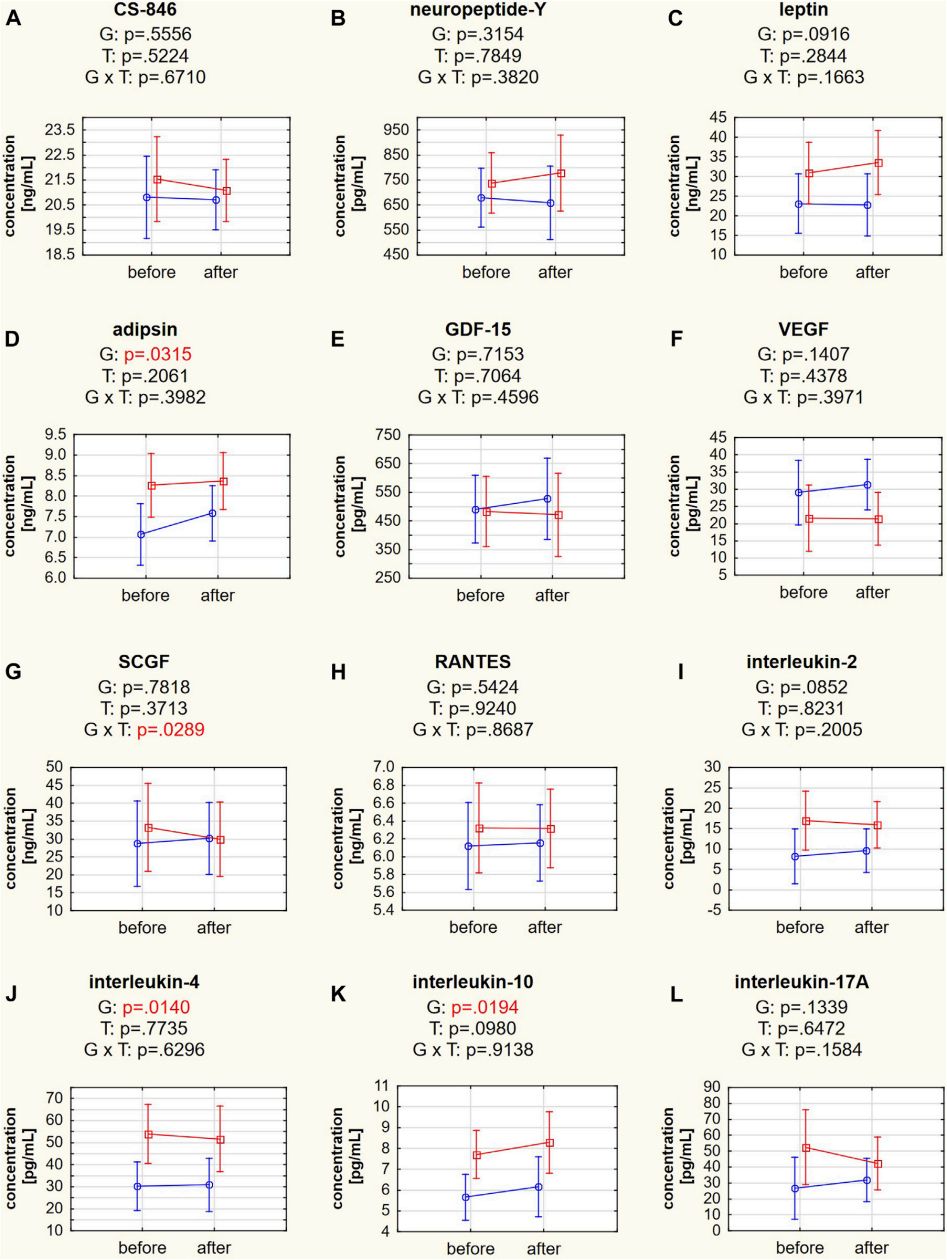
**(A–L)** Biochemical variables in responders (red square-ended line; >50% pain relief) and nonresponders (blue circle-ended line; <50% pain relief) before and after the therapy. G refers to the group effect, T refers to the time effect, and GxT refers to the interaction in a two-way ANOVA.

Adipsin, IL-4, and IL-10 concentrations differed significantly between the responder and nonresponder groups (*post hoc* tests: *p* = 0.0316, *p* = 0.0141, and *p* = 0.0195, respectively) (Figures 5D,J,K). The *post hoc* analysis of the group × time interaction in SCGF concentration did not indicate differences between the studied groups or terms (*post hoc* tests: *p* = 0.1384 for responders and *p* = 0.7505 for nonresponders). However, a Mann-Whitney U test indicated a significant difference in the change in SCGF concentration in the responder and nonresponder groups (*p* = 0.0418). The SCGF concentration decreased in the responder group (−3.24 ng/mL) but increased in the nonresponder group (+1.41 ng/mL). ANOVA showed no significant differences between groups, changes over time, or interactions for the other biochemical variables (Figures 5A–C,E–G,I,L, Supplementary Table S3).

**FIGURE 6:**
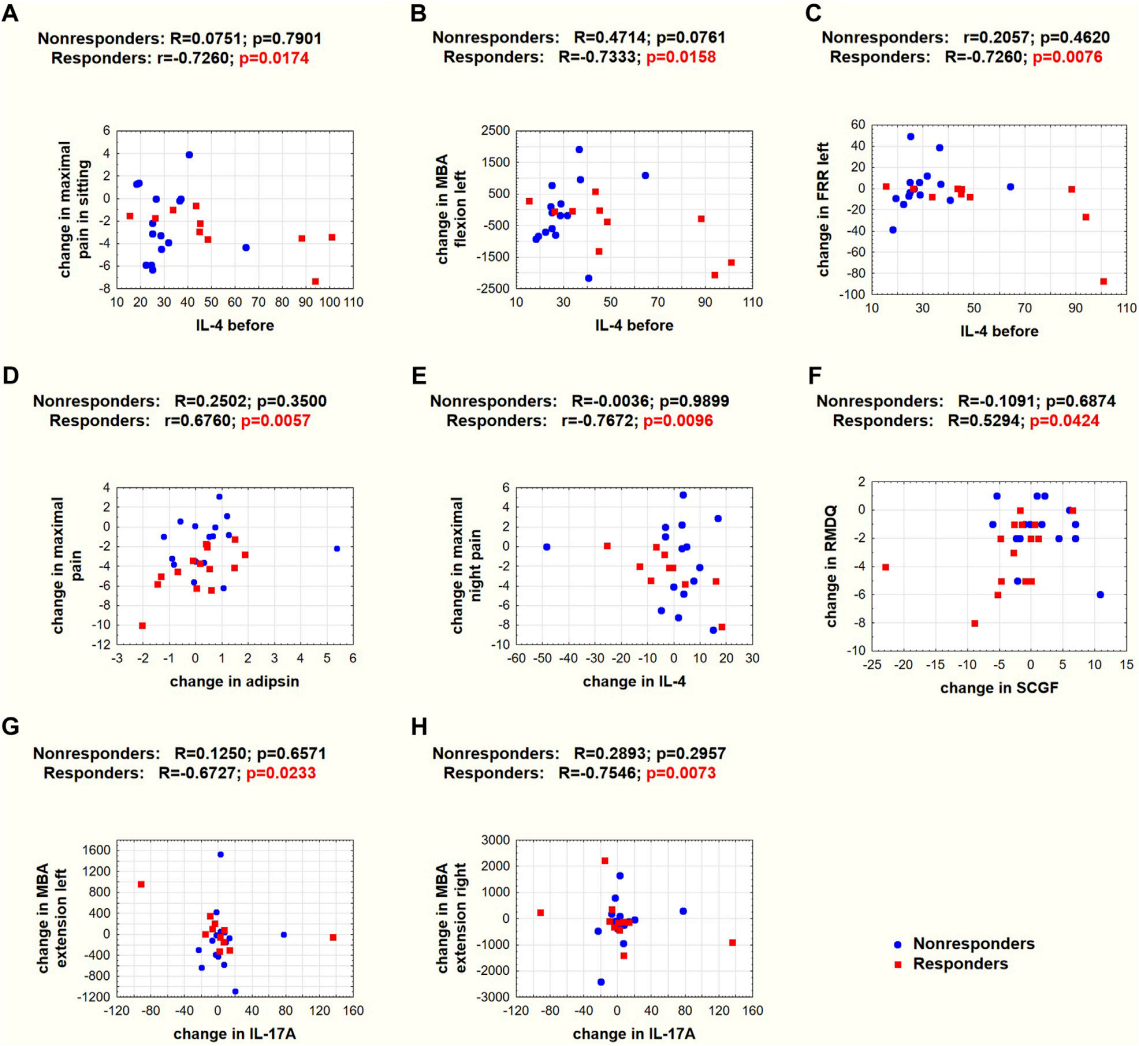
Spearman’s (R) and Pearson’s (r) correlation coefficients and *p* values for selected correlations. **(A–C)** Correlations between baseline IL-4 concentrations and changes in pain and SEMG variables. **(D–H)** Correlations between systemic mediator concentration changes and changes in pain on a VAS scale, RMDQ, or SEMG variables. Red squares denote responders, and blue circles denote nonresponders.

Those patients with a higher IL-4 concentration before therapy achieved a greater reduction in maximal pain on a VAS in the sitting position (Figure 6A), bioelectrical muscle activity in flexion (Figure 6B), and FRR (Figure 6C) on the left side of the body.

In the responder group, the greater the decrease in adipsin concentration, the greater the reduction in maximal pain on a VAS (Figure 6D); the smaller the decrease or greater the increase in IL-4 concentration, the greater the reduction in night pain on a VAS (Figure 6E); the greater the decrease in SCGF, the greater the reduction in RMDQ (Figure 6F); and the greater the decrease in IL-17A concentration, the greater the increase in MBA in extension on the left (Figure 6G) and right (Figure 6H) sides of the spine.

## Discussion

The study indicated that while the examined patients did not differ before therapy in terms of age, body composition, pain intensity, disability, or the degree of degeneration of the lumbar discs, those who benefited more from traction therapy were characterized by a higher content of pro- and anti-inflammatory cytokines, higher FER in the flexion-extension task, and a lower SEMG signal during extension (Table 1). These results suggest that some of the measured variables might have a predictive value for the effectiveness of the applied therapy.

Traction therapy is recommended when back pain is caused by degenerative disk disease with herniation or spinal stenosis and for facet joint degenerations, connective tissue changes, muscle contractures (Pellecchia, 1994), and radiculopathy (Moustafa and Diab, 2013). Mechanical traction can effectively reduce LBP (Cheng et al., 2020) and improve ODI in patients with lumbar disc herniation (Wang et al., 2022). Masood et al. (Masood et al., 2022) suggested that simultaneous reductions in pain and disability levels are possible only when relatively high traction forces are applied. In our study, the traction force was targeted at 30% of body weight, which turned out to be a sufficient level to reduce pain (in four different categories) and the disability level measured by ODI and RMDQ (Figure 3). However, it is unlikely that the response to traction therapy depends on the pain or disability level since responders and nonresponders had similar pain levels on a VAS and disability indices at baseline (Table 1).

### Surface electromyography biomarkers of chronic low back pain

Traction mechanisms to relieve pain result from separating the vertebrae, removing pressure or contact forces from injured tissues, increasing peripheral circulation, and reducing muscle spasm (Krause et al., 2000). Our study did not directly investigate these changes, but we measured variables indirectly related to changes in muscle function. SEMG makes it possible to distinguish individuals with and without LBP. Moreover, many patients with LBP categorized as nonspecific have musculoskeletal abnormalities, highlighting the importance of this type of examination. The flexion-extension task used in our study is based on the lumbar erector spinae flexion-relaxation phenomenon, in which the lumbar muscles relax completely during maximum voluntary flexion. This phenomenon can be observed in most LBP-free subjects but is often absent in patients with chronic LBP (Neblett et al., 2013). They cannot relax their spinal extensor muscles at the end range of lumbopelvic flexion (Shigetoh et al., 2020), which may be related to fear of pain-promoting muscle guarding during flexion (Watson et al., 1997).

It has been shown that FRR and FER measured during SEMG examination are sensitive tools to indicate improvement in muscle function (Mayer et al., 2009). The MBA in a fully flexed position is significantly greater in subjects with than without chronic LBP. Consequently, the FRR is lower in chronic LBP patients than in the control group (Watson et al., 1997). The cutoff between subjects with and without chronic LBP has been proposed as ≥9.5 (Neblett et al., 2013), suggesting that not all our patients had abnormal muscle activity. A recent clinical study determined that the positive influence of 12-week physical training on FRR in patients with LBP was mainly due to reductions in MBA in the relaxation phase (Marshall and Murphy, 2006). At the beginning of our study, responders and nonresponders did not differ significantly in FRR or its components. Moreover, FRR also did not change after therapy, although its component, MBA in flexion on the right side, was the only SEMG variable that changed after therapy (Figure 4).

However, the FER is calculated based on a concentric phase of muscle activity (the MBA in relaxation is divided by the MBA value during extension). Therefore, this ratio is close to zero in individuals with normal relaxation. The higher the ratio, the greater the reduction in the flexion-relaxation phenomenon and the likelihood of LBP (Laird et al., 2019). In our study, FER was significantly lower in nonresponders than in responders at baseline. At the same time, the FER component, the MBA during extension on the left side, was higher in nonresponders (Table 1). Shirado et al., 1995 reported that LBP patients tended to have lower muscle electromyographic activity in extension than healthy controls. These results suggest that the cause of the back pain in women who improved due to therapy (responders) might be more related to impaired neuromuscular activation, directly or indirectly, since some abnormalities subsequently cause muscle dysfunction. However, Wei et al., 2019 showed that symptomatic subjects had higher SEMGs during extension. Moreover, the mean FERs of patients with chronic nonspecific LBP were considerably higher (0.90 in patients with LBP and 0.47 in pain-free controls) than in our study in which all patients had LBP, but the mean FER did not reach the cutoff of 0.692 in any group. This difference would indicate less severe muscle dysfunction.

In summary, beneficiaries of lumbar traction are those patients with more serious muscle dysfunction, which contradicts studies on standard exercise therapy and mobilization techniques, in which patients with lower RMS discrepancies in SEMG topography from healthy individuals were more likely to recover (Hu et al., 2014). In addition, Neblett et al., 2003 concluded that healthier individuals were more likely to benefit from tertiary functional restoration rehabilitation (Neblett et al., 2003).

### Systemic biomarkers of chronic low back pain

Systemic biomarkers are also important factors in evaluating LBP progression (Weber et al., 2015; Wang et al., 2016; Tarabeih et al., 2020), and there has been an attempt to assess their prognostic usefulness in LBP treatment (Schaaf et al., 2021). In our study, we focused on twelve biochemical variables. Initially, the responders differed from the nonresponders in the concentrations of four tested biomarkers: adipsin, proinflammatory IL-2, and anti-inflammatory IL-4 and IL-10 (Table 1).

Adipsin is an adipokine with proinflammatory properties (Fantuzzi, 2005) and is thought to be related to the pathogenesis of back pain through the metabolic pathway. Despite a tendency for higher adipsin concentrations in individuals with increased fat content (Napolitano et al., 1994), adipsin concentrations are higher in those with LBP, even after adjusting for BMI, waist circumference, and fat mass (Brady et al., 2018).

Interleukins act as means of communication for innate and adaptive immune cells and non-immune cells and tissues (Xu et al., 2022). They are secreted mainly by activated T cells (Liao et al., 2011). IL-2 has been classified as proinflammatory for years because it activates pathways leading to T-cell proliferation, survival, and cytokine production. It was recently discovered that IL-2 also promotes the suppression of inflammatory responses through a negative feedback mechanism involving IL-2 receptor internalization, cell death induction, and activation and production of regulatory T lymphocytes (Lan et al., 2008).

While anti-inflammatory cytokines, such as IL-4 and IL-10, are produced mainly by T helper 2 (Th2) lymphocytes and B cells, an additional and important source of IL-10 may be activated macrophages and monocytes. Two anti-inflammatory roles of IL-4 are promoting Th2 lymphocyte development and inhibiting some proinflammatory cytokine synthesis. In addition, IL-10 inhibits cytokine production in monocytes and neutrophils and inhibits T-helper 1-type lymphocytes (Opal and DePalo, 2000). The interactions between proinflammatory and anti-inflammatory molecules are complex under physiologic or pathologic conditions. Physiologically, they limit the injurious effects of prolonged or overactive inflammatory reactions. However, in pathological conditions, the control of anti-inflammatory mediators may be insufficient to overcome proinflammatory activities, or inversely, an overcompensation of their secretion may inhibit the immune response, rendering the host at risk of systemic inflammation (Opal and DePalo, 2000).

In our study, all four biomarkers were present at significantly higher concentrations in responders than in nonresponders (Table 1). Weber et al., 2015 demonstrated that subjects who had LBP due the spinal stenosis and degenerative disc disease had significantly higher IL-2 concentrations than those with LBP due to a disk herniation, and their IL-2 level decreased significantly during treatment with epidural steroid injection (ESI). Moreover, while IL-10 is an anti-inflammatory cytokine, a reduction in IL-10 concentration during therapy correlated with a reduction in pain intensity. In a cohort study on patients with lumbar radicular pain, IL-4 and IL-10 concentrations were higher in patients with mild and severe sciatica than in healthy controls, and interestingly, patients with mild sciatica had higher IL-10 levels than patients with severe sciatica (Wang et al., 2016; Wang et al., 2016)

The traction therapy applied in our study did not reduce the concentration of any studied mediators. However, it should be noted that blood sampling took place shortly after the end of the therapy, and it is unknown whether such changes would occur longer after therapy. Nevertheless, interesting correlations were observed in the responder group. The baseline IL-4 concentration determined the direction and magnitude of change in the intensity of maximal LBP pain on a VAS in the sitting position (Figure 5A) and the MBA of muscles during flexion (Figure 5B) and FRR (Figure 5C) on the left side in the SEMG test. These observations are valuable since they relate the same systemic biomarker with subjective pain intensity and objective changes in muscle bioelectrical activity. In addition, there was a positive relationship between changes in adipsin concentration and maximal LBP pain on a VAS (Figure 5D). Moreover, there was a negative relationship between changes in IL-4 concentration and maximal night LBP on a VAS (Figure 5E). To our knowledge, this is the first study to demonstrate that changes in systemic IL-4 levels are negatively correlated with changes in the functional status of patients with LBP. These observations reinforce the belief in the protective role of IL-4 in the course of LBP.

Recent findings implicate another factor related to the communication of inflammatory mediators. Teodorczyk-Injeyan et al. (Teodorczyk-Injeyan et al., 2019) indicated a significant disturbance in the IL-6/IL-10 ratio in patients with chronic LBP and the IL-2/IL-10 ratio in patients with acute LBP relative to control healthy subjects. These findings suggest that a balance between proinflammatory and anti-inflammatory cytokines may be more informative than the concentrations of individual mediators. An imbalance between proinflammatory and anti-inflammatory mediators has already been suggested to contribute to the pathophysiology of LBP (Li et al., 2016) and degenerative joint disease (Wojdasiewicz et al., 2014). In our study, IL-2/IL-10 ratio did not differ between subjects. Nevertheless, these are data only from this one indicator and not the overall ratio of inflammatory to anti-inflammatory cytokines, so it is worth examining a wider range of cytokines in the future.

T helper 17 lymphocytes and IL-17 are involved in the pathomechanism of LBP in patients with lumbar disk herniation in an autoimmune manner, regulating inflammation, chemotaxis, and angiogenesis during the healing process (Tian et al., 2015). In our study, the IL-17A concentration did not differ between responders and nonresponders (Table 1), and we did not observe a post-treatment decrease (Figure 5L). However, a significant correlation was found between changes in IL-17A concentration and maximal muscle activity in the extension phase of the SEMG test on both sides of the body (Figures 6G,H). This novel finding confirms that a change in inflammatory status significantly affects the functioning of patients with LBP. High IL-17 concentrations were previously associated with lumbar disk herniation, especially when the annulus fibrosus was ruptured (Tian et al., 2015). The IL-17 concentration was also reduced after applying ESI in patients with LBP diagnosed with disk herniation (Weber et al., 2015).

Stem cell growth factor (SCGF/C-type lectin domain containing 11A [CLEC11a]) was found to be an essential growth factor that promotes osteogenesis and is required for skeletal maintenance. SCGF was also necessary for normal fracture healing (Yue et al., 2016). The opposite direction of SCGF changes between responders and nonresponders was observed in our study (Figure 5G). However, the SCGF level did not differentiate patients before the therapy (Table 1). Previous studies showed reduced SCGF levels in patients with LBP after ESI treatment, particularly in patients with spinal stenosis and degenerative disc disease (Weber et al., 2015). SCGF correlated positively with changes in pain levels in patients with LBP due to disk herniation but not in patients with spinal stenosis and degenerative disk disease. Therefore, the tendency of reduced SCGF concentrations in the responder group could also be considered a positive effect of the traction therapy, especially since this is consistent with a positive correlation between changes in SCGF and disability scores in the RMDQ in the responder group (Figure 6F). However, in our cohort treated with traction forces, we failed to find a relationship between SCGF and pain intensity.

No significant changes due to traction therapy nor between responders and nonresponders at baseline were found in the concentrations of other biomarkers measured in our study: CS-846, neuropeptide-Y, leptin, GDF-15, VEGF, and RANTES. However, we cannot rule them out as biomarkers of potential prognostic value since they have numerous reported associations with LBP or the degree of spinal degeneration (Tian et al., 2015; Weber et al., 2015; Oldenburg et al., 2016; Schaaf et al., 2021).

Some of the examined biomarkers may be useful in the future to predict improved pain response and pain-related function. However, their sensitivity will likely depend on the etiology of the LBP and the type of therapy applied. This conclusion also points to some limitations of this study and indications for further studies. Firstly, blood sample examinations should occur more than once after therapy to show the complete picture of changes in cellular inflammatory and metabolic processes seen in systemic biomarker concentrations. It is important to capture the moment the subject returns to homeostasis after the intervention. Secondly, future studies should examine more mediators and perhaps include tests intended for clinical diagnostics to assess whether the values exceed the reference values for inflammation. It should be noticed that although the examinations were performed in every woman after approximately 30 days, we were not sure whether they were examined in the same phase of the menstrual cycle, and this might influence the perception of pain. Finally, due to the high variability of the studied variables, the sample size should be increased to increase the effect size and allow for more reliable conclusions. Nevertheless, the strength of this study was its application of restrictive exclusion criteria in the admission of participants into the study, which allowed us to generate questions and hypotheses for further studies.

## Conclusion

We determined the phenotype of patients who benefit most from lumbar traction therapy. Our study showed that women who had significantly reduced pain due to traction therapy had SEMG indices of less favorable muscle activity in the flexion-extension task and elevated indices of inflammation before the study. Changes in adipsin, SCGF, and IL-17A concentrations were associated with changes in pain intensity, disability level, and muscle activity, respectively. For the first time, we added IL-4 to the list of potential biomarkers for prognosing post-therapy changes in pain intensity and muscle activity. However, research on a larger sample size should be performed. Collecting information on possible biomarkers for pain and its related disability will enable the development of a prognostic tool to provide valuable predictions to aid in selecting the most appropriate treatment for different etiological types of LBP. It would be a significant advance in physiotherapy if it were possible to estimate the likelihood of improvement in a patient undergoing physical or manual therapy and to prescribe the intervention only to those most likely to benefit from it.

## Data Availability

The raw data supporting the conclusion of this article will be made available by the authors, without undue reservation.

## Abbreviations

CS846, aggrecan chondroitin sulfate 846 epitope; ESI, epidural steroid injection; FER, flexion extension ratio; FRR, flexion relaxation ratio; GDF-15, growth and differentiation factor 15; IL-2, interleukin-2; IL-4, interleukin-4; IL-10, interleukin-10; IL-17A, interleukin-17A; LBP, low back pain; MBA, maximal bioelectrical activity of the longissimus muscle; ODI, Oswestry disability index; RANTES, regulated on activation in normal t-cell expressed and secreted; RMDQ, Roland Morris Disability Questionnaire; RMS, root mean square; SCGF, stem cell growth factor; SEMG, surface electromyography; Th2, T helper 2; VAS, visual analogue scale; VEGF-A, vascular endothelial growth factor A.
