# Supplementary material for: In search of biomarkers for low back pain: can traction therapy effectiveness be prognosed by surface electromyography or blood parameters?
Source: Front Physiol. 2023 Dec 8;14:1290409. doi: 10.3389/fphys.2023.1290409 (PMC10739392; doi:10.3389/fphys.2023.1290409)
Supplement: Supplementary file 1 [file Table1.DOCX]

**Table S1.** Summary ANOVA results on effects of traction therapy- subjective variables

|  | | **Responders** | **Nonresponders** | **ANOVA main effects** | | |
| --- | --- | --- | --- | --- | --- | --- |
|  |  | **MEAN (SD) (n=15)** | **MEAN (SD) (N=16)** | **Group effect**  p value  (ƞ^2^) | **Time effect**  p value  (ƞ^2^) | **Group x Time**  p value  (ƞ^2^) |
| Maximal LBP  [VAS 0-10] | **PRE** | 5.08 (2.44) | 5.96 (2.66) | **0.0067**  **(0.23)** | **0.0000**  **(0.61)** | **0.0072**  **(0.22)** |
|  | **POST** | 0.89 (0.70) | 4.28 (2.92) |  |  |  |
| Maximal morning LBP [VAS 0-10] | **PRE** | 4.35 (3.01) | 3.98 (3.19) | 0.7312  (0.00) | **0.0000**  **(0.51)** | 0.1833  (0.06) |
|  | **POST** | 1.03 (1.06) | 1.99 (3.00) |  |  |  |
| Maximal night LBP  [VAS 0-10] | **PRE** | 2.98 (2.39) | 3.31 (3.47) | 0.1961  (0.06) | **0.0014**  **(0.30)** | 0.3149  (0.04) |
|  | **POST** | 0.32 (0.55) | 1.84 (2.69) |  |  |  |
| Maximal LBP at the sitting position [VAS 0-10] | **PRE** | 4.09 (2.27) | 4.77 (2.71) | 0.0728  (0.11) | **0.0000**  **(0.48)** | 0.2785  (0.04) |
|  | **POST** | 0.91 (0.99) | 2.70 (2.78) |  |  |  |
| RMDQ  [0-24] | **PRE** | 4.67 (3.62) | 4.31 (2.89) | 0.5997  (0.01) | **0.0000**  **(0.50)** | **0.0353**  **(0.14)** |
|  | **POST** | 1.67 (2.13) | 3.06 (2.95) |  |  |  |
| Oswestry Disability Index [0-50] | **PRE** | 11.93 (5.99) | 12.06 (5.59) | 0.1973  (0.06) | **0.0000**  **(0.52)** | **0.0192**  **(0.18)** |
|  | **POST** | 4.93 (4.43) | 9.38 (5.41) |  |  |  |
| Bold indicate significant (p < 0.05)  RMDQ: Roland-Morris Disability Questionnaire | | | | | | |
