# Supplementary material for: In search of biomarkers for low back pain: can traction therapy effectiveness be prognosed by surface electromyography or blood parameters?
Source: Front Physiol. 2023 Dec 8;14:1290409. doi: 10.3389/fphys.2023.1290409 (PMC10739392; doi:10.3389/fphys.2023.1290409)
Supplement: Supplementary file 2 [file Table2.DOCX]

|  | | **Responders** | **Nonresponders** | **ANOVA main effects** | | |
| --- | --- | --- | --- | --- | --- | --- |
|  |  | **MEAN (SD) (n=15)** | **MEAN (SD) (N=16)** | **Group effect**  p value  (ƞ^2^) | **Time effect**  p value  (ƞ^2^) | **Group x Time**  p value  (ƞ^2^) |
| MBA flexion – L (μV) | **PRE** | 1195 (818) | 1235 (777) | 0.9601  (0.00) | 0.3598  (0.03) | 0.8809  (0.00) |
|  | **POST** | 1040 (688) | 1019 (784) |  |  |  |
| MBA flexion – R (μV) | **PRE** | 1427 (811) | 1274 (683) | 0.4463  (0.02) | **0.0231**  **(0.17)** | 0.9396  (0.00) |
|  | **POST** | 1018 (642) | 890 (589) |  |  |  |
| MBA relaxation – L (μV) | **PRE** | 241 (173) | 184 (157) | 0.4431  (0.02) | 0.3864  (0.03) | 0.5742  (0.01) |
|  | **POST** | 204 (168) | 176 (173) |  |  |  |
| MBA relaxation – R (μV) | **PRE** | 236 (151) | 171 (148) | 0.2525  (0.05) | 0.2798  (0.04) | 0.9178  (0.00) |
|  | **POST** | 275 (213) | 217 (208) |  |  |  |
| MBA extension - L (μV) | **PRE** | 997 (829) | 1262 (338) | 0.1332  (0.08) | 0.4539  (0.02) | 0.8247  (0.00) |
|  | **POST** | 923 (341) | 1124 (634) |  |  |  |
| MBA extension - R (μV) | **PRE** | 981 (480) | 1252 (627) | 0.1738  (0.06) | 0.6190  (0.01) | 0.6217  (0.01) |
|  | **POST** | 981 (599) | 1106 (540) |  |  |  |
| FRR – L | **PRE** | 17.53 (25.63) | 15.01 (10.85) | 0.8547  (0.00) | 0.5227  (0.01) | 0.4351  (0.02) |
|  | **POST** | 11.43 (9.61) | 15.62 (18.24) |  |  |  |
| FRR – R | **PRE** | 17.36 (14.77) | 31.52 (49.11) | 0.2758  (0.04) | 0.0624  (0.12) | 0.3438  (0.03) |
|  | **POST** | 11.21 (8.62) | 13.21 (9.57) |  |  |  |
| FER - left side | **PRE** | 0.31 (0.25) | 0.17 (0.17) | 0.1606  (0.07) | 0.9893  (0.00) | 0.2875  (0.04) |
|  | **POST** | 0.26 (0.21) | 0.22 (0.24) |  |  |  |
| FER - right side | **PRE** | 0.27 (0.18) | 0.17 (0.20) | 0.2235  (0.05) | 0.2052  (0.06) | 0.7113  (0.01) |
|  | **POST** | 0.31 (0.23) | 0.25 (0.27) |  |  |  |
| Bold indicate significant (p < 0.05),  MBA: maximal bioelectrical activity of the longissimus muscle, FRR: flexion relaxation ratio, FER: flexion extension ratio, L:left side, R:right side | | | | | | |

**Table S2.** Summary ANOVA results on effects of traction therapy- EMG variables
