# Supplementary material for: In search of biomarkers for low back pain: can traction therapy effectiveness be prognosed by surface electromyography or blood parameters?
Source: Front Physiol. 2023 Dec 8;14:1290409. doi: 10.3389/fphys.2023.1290409 (PMC10739392; doi:10.3389/fphys.2023.1290409)
Supplement: Supplementary file 3 [file Table3.DOCX]

**Table S3.** Summary ANOVA results on effects of traction therapy- biochemical variables

|  | | **Responders** | **Nonresponders** | **ANOVA main effects** | | |
| --- | --- | --- | --- | --- | --- | --- |
|  |  | **MEAN (SD) (n=15)** | **MEAN (SD) (N=16)** | **Group effect**  p value  (ƞ^2^) | **Time effect**  p value  (ƞ^2^) | **Group x Time**  p value  (ƞ^2^) |
| CS846 [ng/mL] | **PRE** | 21.54 (3.56) | 20.81 (2.85) | 0.5556  (0.02) | 0.5224  (0.01) | 0.6710  (0.01) |
|  | **POST** | 21.09 (2.54) | 20.72 (2.18) |  |  |  |
| NPY [pg/mL] | **PRE** | 738.14 (253.41) | 680.28 (204.84) | 0.3154  (0.04) | 0.7849  (0.00) | 0.3820  (0.03) |
|  | **POST** | 778.69 (361.28) | 658.93 (197.09) |  |  |  |
| Leptin [ng/mL] | **PRE** | 30.89 (16.44) | 23.08 (13.28) | 0.0916  (0.10) | 0.2844  (0.04) | 0.1663  (0.07) |
|  | **POST** | 33.56 (17.26) | 22.73 (13.44) |  |  |  |
| Adipsin [ng/mL] | **PRE** | 8.26 (1.23) | 7.07 (1.65) | **0.0315**  (0.15) | 0.2061  (0.06) | 0.3982  (0.03) |
|  | **POST** | 8.37 (1.13) | 7.58 (1.47) |  |  |  |
| GDF-15 [pg/mL] | **PRE** | 483.48 (222.58) | 490.93 (240.61) | 0.7153  (0.01) | 0.7064  (0.01) | 0.4596  (0.02) |
|  | **POST** | 471.57 (192.60) | 527.38 (336.37) |  |  |  |
| VEGF A [pg/mL] | **PRE** | 21.54 (5.32) | 29.03 (24.87) | 0.1407  (0.07) | 0.4378  (0.02) | 0.3971  (0.03) |
|  | **POST** | 21.44 (5.55) | 31.36 (19.35) |  |  |  |
| SCGF [ng/mL] | **PRE** | 33.23 (32.76) | 28.76 (6.95) | 0.7818  (0.00) | 0.3713  (0.03) | **0.0289**  (0.15) |
|  | **POST** | 29.99 (27.54) | 30.16 (6.17) |  |  |  |
| RANTES [ng/mL] | **PRE** | 6.32 (1.26) | 6.12 (0.53) | 0.5424  (0.01) | 0.9240  (0.00) | 0.8687  (0.00) |
|  | **POST** | 6.32 (1.12) | 6.15 (0.43) |  |  |  |
| Interleukin-2 [pg/mL] | **PRE** | 16.96 (18.72) | 8.15 (4.14) | 0.0852  (0.10) | 0.8231  (0.00) | 0.2005  (0.06) |
|  | **POST** | 15.93 (14.43) | 9.62 (4.93) |  |  |  |
| Interleukin-4 [pg/mL] | **PRE** | 53.89 (29.73) | 30.21 (11.41) | **0.0140**  (0.24) | 0.7735  (0.00) | 0.6296  (0.01) |
|  | **POST** | 60.77 (44.57) | 30.78 (9.28) |  |  |  |
| Interleukin-10 [pg/mL] | **PRE** | 7.72 (2.25) | 5.66 (2.10) | **0.0194**  (0.18) | 0.0980  (0.09) | 0.9137  (0.00) |
|  | **POST** | 8.29 (3.21) | 6.16 (2.38) |  |  |  |
| Interleukin-17A [pg/mL] | **PRE** | 52.48 (55.97) | 26.69 (11.78) | 0.1339  (0.10) | 0.6472  (0.01) | 0.1584  (0.09) |
|  | **POST** | 50.79 (40.12) | 31.95 (21.98) |  |  |  |
| IL-2/IL-10 ratio | **PRE** | 2.46 (3.68) | 1.61 (1.11) | 0.4832  (0.02) | 0.8729  (0.00) | 0.1072  (0.09) |
|  | **POST** | 2.20 (2.77) | 1.82 (1.24) |  |  |  |
| Bold indicate significant (p < 0.05)  CS846: aggrecan chondroitin sulfate 846 epitope, GDF-15: growth and differentiation factor 15, VEGF: vascular endothelial growth factor, SCGF: stem cell growth factor, RANTES: regulated on activation, normal T-cell expressed and secreted; IL-2/IL-10 ratio: interleukin 2 to interleukin-10 ratio | | | | | | |
